# Effects of different carbohydrate sources on fructan metabolism in plants of *Chrysolaena obovata* grown *in vitro*

**DOI:** 10.3389/fpls.2015.00681

**Published:** 2015-09-07

**Authors:** Flavio Trevisan, Vanessa F. Oliveira, Maria A. M. Carvalho, Marília Gaspar

**Affiliations:** ^1^Núcleo de Pesquisa em Fisiologia e Bioquímica, Instituto de BotânicaSão Paulo, Brazil; ^2^Instituto Federal de São Paulo, Campus São RoqueSão Roque, Brazil; ^3^Núcleo de Ciências da Saúde, Universidade de Mogi das CruzesSão Paulo, Brazil

**Keywords:** inulin, plant tissue culture, 1-SST, 1-FFT, 1-FEH, sucrose, sugar modulation, relative expression

## Abstract

*Chrysolaena obovata* (Less.) Dematt., previously named *Vernonia herbacea*, is an Asteraceae native to the Cerrado which accumulates about 80% of the rhizophore dry mass as inulin-type fructans. Considering its high inulin production and the wide application of fructans, a protocol for *C. obovata in vitro* culture was recently established. Carbohydrates are essential for *in vitro* growth and development of plants and can also act as signaling molecules involved in cellular adjustments and metabolic regulation. This work aimed to evaluate the effect of different sources of carbohydrate on fructan metabolism in plants grown *in vitro*. For this purpose, *C. obovata* plants cultivated *in vitro* were submitted to carbon deprivation and transferred to MS medium supplemented with sucrose, glucose or fructose. Following, their fructan composition and activity and expression of genes encoding enzymes for fructan synthesis (1-SST and 1-FFT) and degradation (1-FEH) were evaluated. For qRT-PCR analysis partial cDNA sequences corresponding to two different *C. obovata* genes, 1-SST and 1-FFT, were isolated. As expected, *C. obovata* sequences showed highest sequence identity to other Asteraceae 1-SST and 1-FFT, than to Poaceae related proteins. A carbon deficit treatment stimulated the transcription of the gene *1-FEH* and inhibited *1-SST* and *1-FFT* and carbohydrate supplementation promoted reversal of the expression profile of these genes. With the exception of *1-FFT*, a positive correlation between enzyme activity and gene expression was observed. The overall results indicate that sucrose, fructose and glucose act similarly on fructan metabolism and that *1-FEH* and *1-SST* are transcriptionally regulated by sugar in this species. Cultivation of plants in increasing sucrose concentrations stimulated synthesis and inhibited fructan mobilization, and induced a distinct pattern of enzyme activity for 1-SST and 1-FFT, indicating the existence of a mechanism for differential regulation between them.

## Introduction

The Cerrado and its savanna like vegetation is the second largest biome in the Brazilian territory, being outsized only by the Amazon rain forest. It covers approximately 21% of the Brazilian land area and is characterized by a well-defined seasonality, concerning the water regime, which includes a humid summer and a dry winter often lasting up to 5 months (Eiten, 1972; Coutinho, 2002). The presence of fructan accumulating Asteraceae in the Cerrado has been well documented in various studies (Figueiredo-Ribeiro et al., 1986; Tertuliano and Figueiredo-Ribeiro, 1993; Joaquim et al., 2014).

*Chrysolaena obovata* (Less.) Dematt., previously named *Vernonia herbacea* (Asteraceae), is a perennial herb native to and widely spread in the Brazilian Cerrado (Figueiredo-Ribeiro et al., 1986). Seeds of *C. obovata* present a very low germination rate and only 15% of the achenes enclose embryos (Sassaki et al., 1999). Due to limited seed germination, the underground reserve organ, rhizophore, is commonly used for vegetative propagation (Hayashi and Appezzato-da-Glória, 2005). Nonetheless, in a recent study, seeds were successfully used as explant material for the establishment of *in vitro* culture of *C. obovata* (Trevisan et al., 2014). Besides the role in vegetative propagation, the rhizophores accumulate ca. 80% of its dry mass as inulin type fructans (Carvalho and Dietrich, 1993), showing a high potential for inulin production. Previous results showing efficient production of inulin in field trials and *in vitro* by *C. obovata* (Carvalho et al., 1998; Trevisan et al., 2014) suggest that this native species could be a promising alternative for fructan production, since inulin for commercial use is mainly extracted from roots of *Chichorium intybus* L.

Inulin is synthesized from sucrose by the action of fructosyltransferases. The first enzyme, sucrose:sucrose fructosyltransferase (1-SST, EC 2.4.1.99), catalyses the production of the trisaccharide 1-kestotriose. The second enzyme, fructan:fructan fructosyltransferase (1-FFT, EC 2.4.1.100), catalyses the reversible transfer of fructosyl units from a fructan molecule with a DP ≥ 3 to another fructan molecule or to sucrose, resulting in fructan chains with a wide range of lengths. Inulin degradation is catalyzed by fructan exohydrolase (1-FEH, EC 3.2.1.153), which releases free fructose from the terminal non-reducing fructosyl unit (Edelman and Jefford, 1968; Carvalho et al., 2007). The gene coding for *C. obovata* 1-FEH has been isolated and its activity on inulin hydrolysis has been confirmed by heterologous expression in *Pichia pastoris* (Asega et al., 2008).

Fructan metabolism is regulated by several endogenous factors such as hormones and sugars (Kusch et al., 2009; Súarez-González et al., 2014; Trevisan et al., 2014), as well as by exogenous factors, such as low temperature, drought, CO_2_ atmospheric concentration (De Roover et al., 1999; Livingston et al., 2009; Oliveira et al., 2010; Asega et al., 2011; Garcia et al., 2011).

Sucrose is able to play double role, serving as substrate for fructan synthesis and as a regulating factor of gene expression (Pollock and Cairns, 1991; Gupta and Kaur, 2005). Fructan synthesis is induced by increasing sucrose concentration in the cell vacuole (Pollock and Cairns, 1991), although other sugars such as glucose and fructose are also able, less effectively than sucrose, to stimulate fructan synthesis in excised leaves of *Dactylis glomerata* (Maleux and Van den Ende, 2007). However, the combination of both hexoses was equally effective, suggesting their quick conversion into sucrose to exert the positive effect on fructan accumulation.

In *Agave tequilana* and *A. inaequidens*, the cultivation of plants in culture media supplemented with 8% sucrose stimulated the expression of the genes *1-SST* and *1-FFT* in leaves and stem, promoting fructan accumulation (Súarez-González et al., 2014). Sucrose was the most efficient elicitor of *1-SST* and *1-FFT* gene expression in the stems of *A. inaequidens* when compared to other treatments known to regulate fructan metabolism, such as abscisic acid, cytokinin, and osmotic stress.

Conversely, Kusch et al. (2009) working with *C. intybus* hairy root cultures, showed that increasing sucrose concentration from 3 to 6% did not result in *1-SST* and *1-FFT* transcript accumulation. However, the increase in transcript and inulin accumulation occurred by lowering nitrogen supply under high sucrose concentration, indicating that sucrose alone was not sufficient to induce inulin synthesis in this system.

Calcium was also shown to be essential to induce the activity and expression of fructosyltransferases mediated by sucrose in wheat leaves (Martínez-Noël et al., 2006), as a component of the sucrose signaling pathway that leads to the induction of fructan synthesis.

Besides stimulating transcription of fructosyltransferases, sucrose inhibits the activity of fructan exohydrolases, as reported for *C. obovata* (Asega et al., 2008), *C. intybus* (Verhaest et al., 2007), *Helianthus tuberosus* (Marx et al., 1997), *Triticum aestivum* (Van den Ende et al., 2003), and *Lolium perenne* (Lothier et al., 2014). Studies with *L. perenne* also analyzed the effect of hexoses, sucrose and their corresponding analogs on FEH activity and fructan mobilization, by spraying defoliated sugar starved plants (Lothier et al., 2010). By these treatments, they showed that sucrose analogs employed (lactulose, palatinose, and turanose) inhibited FEH activity to the same extent as sucrose, suggesting the existence of a sucrose signaling.

In summary, sugar status of plant cells is sensed by sensor proteins, generating signal transduction cascades and influencing the regulation of a large number of genes (Price et al., 2004; Gupta and Kaur, 2005), including those involved in fructan metabolism (Kusch et al., 2009; Súarez-González et al., 2014).

Although the effects of sucrose were described for many fructan accumulating species as reported above, using different experimental systems, these studies focused mainly on the inhibition of fructan exohydrolases activity by sucrose, excepting the work of Martínez-Noël et al. (2006), which focused on the effect of sucrose on fructosyl sucrose-synthesizing enzymes, hampering the discussion about the modulation of the whole fructan metabolism by exogenous sucrose in one single species.

Despite the importance of *C. obovata* as a model species for the study of fructan metabolism and the role of sugars in fructan metabolism regulation, there are no reports on the effect of carbon sources on the regulation of fructan metabolism in this species or in any other tropical inulin-accumulating species. As tissue culture is considered a valuable tool for the study of primary and secondary metabolisms, in this paper we report the use of this technique to evaluate the effect of different carbon sources (sucrose, fructose and glucose) on the modulation of the activity and expression of enzymes involved in fructan synthesis and mobilization in plants of *C. obovata* cultivated *in vitro*. We also report on the effect of increasing sucrose concentrations on fructan accumulation in *in vitro* plants, considering that the control and optimization of *in vitro* culture conditions are important to achieve higher inulin production in the future.

## Materials and methods

### *In vitro* culture of *Chrysolaena obovata*

Seeds (achenes) of *C. obovata* were collected in a preserved Cerrado area in Mogi-Guaçu, State of São Paulo, Brazil (22°18′S, 47°11′W), stored at 4°C and used during 2 months. The seeds were surface sterilized by washing with 70% ethanol for 1 min and 2.5% sodium hypochlorite for 30 min. Following, they were washed three times in sterile distilled water and placed in Petri dishes containing the MS culture medium (Murashige and Skoog, 1962) with half-strength of macronutrients, 3% sucrose and 0.8% agar. The pH of the medium was adjusted to 5.8 before autoclaving for 15 min at 120°C. Subsequently, seeds were incubated at 25°C, under 14-h photoperiod (22.5 μmol m^−2^ s^−1^) (Trevisan et al., 2014). One week after germination, plants were transferred to 300 mL glass bottles containing the same medium, subcultured to fresh medium at 4-week intervals and kept in the same conditions for growth.

Aiming at plant propagation, rhizophores from 12 month-old plants germinated *in vitro* were fragmented (2 × 2 mm) and cultured for 5 months in the same conditions described above. A homogenous batch of plants was obtained for use in all experiments.

### Experiment 1: Effects of different carbon sources on fructan metabolism

Plants were maintained for 7 days in MS culture medium with half-strength of macronutrients, 0.8% agar and free of sugar, until the complete depletion of carbon stocks. They were subsequently transferred to the same medium containing 3% sucrose (Suc), 3% fructose (Fru), or 3% glucose (Glc). Sugar concentration of 3% was previously tested for this species and was considered adequate for *C. obovata* growth, development and fructan metabolism *in vitro* (Trevisan et al., 2014). Samples were collected at day 0 (before transfer—control) and after 1, 2, and 5 days of cultivation. Samples of rhizophores and aerial organs (leaves and stems) were frozen in liquid nitrogen and stored at −80°C until analyses. For experiment evaluation, the parameters analyzed were total fructan content, soluble carbohydrate composition, enzyme activity and gene expression of 1-SST, 1-FFT, and 1-FEH.

### Experiment 2: Effects of different sucrose concentrations on fructan accumulation

Plants were grown for 30 days in MS culture medium with half-strength of macronutrients, 0.8% agar and the following sucrose concentrations 0, 3, 6 and 9%. Samples of rhizophores and aerial organs (leaves and stems) were collected from the culture medium, frozen in liquid nitrogen and stored at −80°C until analyses. For experiment evaluation, the parameters analyzed were total fructan content and activity of 1-SST, 1-FFT, and 1-FEH.

### Soluble carbohydrate analyses

Carbohydrates were extracted from freeze-dried tissue samples as previously described (Carvalho et al., 1998), modified as follows: the ethanol and aqueous extracts, constituting the total soluble carbohydrate extract, were pooled and concentrated under vacuum at 35°C. Free and combined fructose (total fructan) were measured by the ketose-specific modification of the anthrone reaction (Jermyn, 1956), using fructose (SigmaAldrich®) as standard. Soluble carbohydrates were de-ionized through Dowex ion exchange columns (SigmaAldrich®), according to Carvalho and Dietrich (1993) and analyzed by HPAEC/PAD, on ICS3000 Dionex system (Dionex, ThermoScientific, USA) with a CarboPacPA-1 column (2 × 250 mm), according to Oliveira et al. (2013). The retention times of the sample peaks were compared with the reference standards glucose, fructose, sucrose (SigmaAldrich®), 1-kestotriose and 1-kestotetraose, and inulin from tubers of *H. tuberosus*.

### Enzyme extraction and assays

Samples were homogenized in 0.05 M McIlvaine buffer (pH 5.5; 1:1, w/v) containing 2 mM EDTA, 2 mM β-mercaptoethanol, 5 mM ascorbic acid, and 10% PVPP (w/w), as described in Asega and Carvalho (2004). Proteins precipitated with (NH_4_)_2_SO_4_ to a final saturation of 80% were re-suspended at a ratio of ca.10 g fresh mass equivalent per mL in extraction buffer. Enzymes were assayed by incubation of the protein extract with different substrates mixed 1:1 (v/v). The substrates were prepared in 0.05 M McIlvaine buffer pH 4.5 for 1-FEH and pH 5.5 for 1-SST and 1-FFT. The extracts were incubated at 30°C at a final concentration of 0.2 M sucrose for 1-SST activity, 0.2 M 1-kestotriose for 1-FFT activity and 5% inulin from *C. obovata* for 1-FEH activity. Incubation time was 4 h for 1-SST, 2 h for 1-FFT and 3 h for 1-FEH. The reactions were stopped by heating the mixture for 5 min at 100°C. Extraction and assay conditions were optimized to ensure reliable activity measurements. For activity determination, samples of the reaction mixtures were diluted 5-fold in deionized water and analyzed using HPAEC/PAD with a 2 × 250 mm CarboPac PA-1 column on a Dionex system as above. The gradient was established by mixing eluent A (150 mm NaOH) with eluent B (500 mm sodium acetate in 150 mm NaOH) as described in Oliveira et al. (2013). The activities of 1-FEH, 1-SST, and 1-FFT were measured by the quantification of peak areas of reaction products fructose, 1-kestotriose and 1-kestotetraose, respectively, by comparison with the external standards.

### Partial cDNA isolation

Total RNA was extracted from 100 mg of frozen samples, using the TRIzol® reagent (Invitrogen) according to the manufacturer instructions. The concentration and integrity of the RNA samples was assessed by spectrophotometer and 1% agarose/formaldehyde gel electrophoresis. Previous to cDNA synthesis, genomic DNA was removed by treatment with DNAse I (Fermentas). cDNA was synthesized using First Strand cDNA Synthesis Kit (Fermentas), according to the manufacturer instructions. Degenerate primers described in Table S1 were used for 1-SST, 1-FFT, and EF (Elongation 1-alpha fator) isolation. The amplification conditions were the following: 94°C for 2 min, 35 cycles of 94°C for 45 s, 58°C for 1 min, 72°C for 1 min; and a final extension at 72°C for 5 min. The expected size cDNA fragments were purified by PureLink Quick Gel Extraction and PCR Purification Combo Kit (Invitrogen) and sequenced using Big Dye Terminator v.3.1 (Applied Biosystems). *C. obovata* sequences were compared against GenBank using the BLASTN algorithm at the NCBI (National Center for Biotechnology Information; http://www.ncbi.nlm.nih.gov/) to confirm identity.

### Phylogenetic analysis

Phylogenetic analysis was performed based on the alignment of *C. obovata* deduced amino acid partial sequences, along with the sequences of 1-SST, 1-FFT, 1-FEH and invertases from Asteraceae, Poaceae and other fructan accumulating species. Sequences were selected from GenBank (access numbers described in Figure legend). The alignment was performed using Clustal X 2.0 (Larkin et al., 2007). Distance analysis was performed by neighbor-joining algorithm using the software MEGA 4 (Tamura et al., 2007). Bootstrap analysis was conducted with 1000 replicates and only the bootstrap values of >70% were considered for the development of the unrooted tree. The tree was redrawn with the software FigTree 1.4.2 (Rambaut, 2014).

### Primer design and validation

Primers for fructan-related and reference genes were designed with the help of Primer 3 Plus software (Rozen and Skaletsky, 2000). For reference gene 18S ribosomal RNA (*18S*) and *1-FEH*, primers were designed in the sequences previously isolated for *C. intybus* and *C. obovata*, respectively (Asega et al., 2008; Maroufi et al., 2010). For *1-SST, 1-FFT* and the reference gene elongation factor 1-alpha (*EF*) primers were chosen on the partial cDNA sequences isolated in this work (Table 1). The amplification of expected PCR products was confirmed by fragment length on 2% agarose gel electrophoresis and sequencing. qRT-PCR amplification efficiencies were calculated for each primer based on a standard curve obtained from tenfold dilution series of a cDNA pool of all tested samples.

**Table 1:** **Primers used for ***C. obovata*** qRT-PCR expression analysis**.

**Gene**	**Primer sequence**	**Fragment size (bp)**	**Tm (°C)**
1-SST	F 5′-CATGCTCTACACTGGCAACG-3′	163	61
	R 5′-TAGATGGGTCCCGAAAATCC-3′		60
1-FFT	F 5′-TGCGATTACGGAAGGTTCTT-3′	140	60
	R 5′-CAACATTATAGATTGTAGCCCATCC-3′		60
1-FEH	F 5′-GGCGGATGTTACAATCTCGT-3′	199	60
	R 5′-GTTTTGGAACACCCGAAAGA-3′		60
EF	F 5′-GCTCCTGGACATCGTGACTT-3′	163	60
	R 5′-GACCCCAAGAGTGAAAGCAA-3′		60
18S	F 5′-GGCGACGCATCATTCAAAT-3′	102	62
	R 5′-TCCGGAATCGAACCCTAAT-3′		59

### Gene expression analysis by qRT-PCR

Total RNA isolation and purification was performed as described earlier. Genomic DNA contamination was removed by treatment with DNAse I (Fermentas). cDNA was synthesized from 1.27 μg of total RNA using the SuperScript VILO cDNA Synthesis Kit (Life Technologies) in a final volume of 20 μL, according to the manufacturer's instructions. The cDNA synthesized was then diluted 1:100 and used as a template for qRT-PCR analyses. A NRT control (not reverse transcribed sample) was also amplified to confirm the absence of genomic DNA contamination. Amplifications were carried out in total volume of 20 uL with EXPRESS SYBR GreenER qPCR SuperMix Kit (Life Technologies) on Mastercycler® ep Realplex 2S (Eppendorf, Hamburg, Germany). PCR conditions used consisted of an initial heating step at 50°C for 2 min, followed by 94°C for 2 min,45 cycles of 94°C for 15 s, 55°C for 1 min. After cycling, melting curves were run from 60°C to 95°C for 20 min, to confirm that a single PCR product was amplified. Results were normalized using *18S* and *EF* as reference genes. The analyses of expression stability of the reference genes were performed with BestKeeper (Pfaffl et al., 2004). Stability values of 0.822 (*p* = 0.004) and 0.872 (*p* = 0.001) were obtained for *18S* and *EF* reference genes, respectively. The relative expression level of target genes was calculated as described by Pfaffl (2001). Values represented the average of two biological replicates with four technical replicates.

### Experimental design and statistical analyses

Trials were set up in a completely randomized experimental design. For experiment 1 (Effects of different carbon sources on fructan metabolism), 3 replicates were used, and for experiment 2 (Effects of different sucrose concentrations on fructan accumulation), 4 replicates were used. In both experiments, each replicate consisted of three plants cultivated in a 300 mL glass bottle, containing 50 mL of culture medium. Data was analyzed by ANOVA with a posteriori comparison of the means using Tukey Honestly Significant Difference procedure, to identify significant differences between treatments. Significant effects are reported at *P* < 0.05.

## Results

### Isolation of the partial cDNAs

Two partial cDNAs coding for the orthologs *1-SST* (accession n° KM597067) and *1-FFT* (KM597068) of *C. obovata* were isolated. The partial coding region of the ortholog of elongation factor 1-alpha (*EF*) (KM597066) was also cloned to allow designing specific primers for gene expression analysis. The *1-FFT* cDNA is 531-bp in length and contains part of the coding region, including the N-terminal region from the domain of glycosyl hydrolases family 32 (GH32). Blast analysis showed that *C. obovata* 1-FFT has 88% amino acid sequence identity with *Cynara scolymus* (AJ000481), 83% with *C. intybus* (AAD00558) and 84% with *H. tuberosus* (CAA0881) 1-FFT sequences, all belonging to the family Asteraceae.

The 390-bp *1-SST* cDNA comprises part of the coding sequence that also includes part of the GH32 domain. The 1-SST sequence showed the highest amino acid sequence identity with 1-SSTs previously isolated from other Asteraceae (94% with *C. intybus*, AAB58909; 93% with *Lactuca sativa*, ABX90019; 93% with *C. scolymus*, CAA70855).

**Figure 1:**
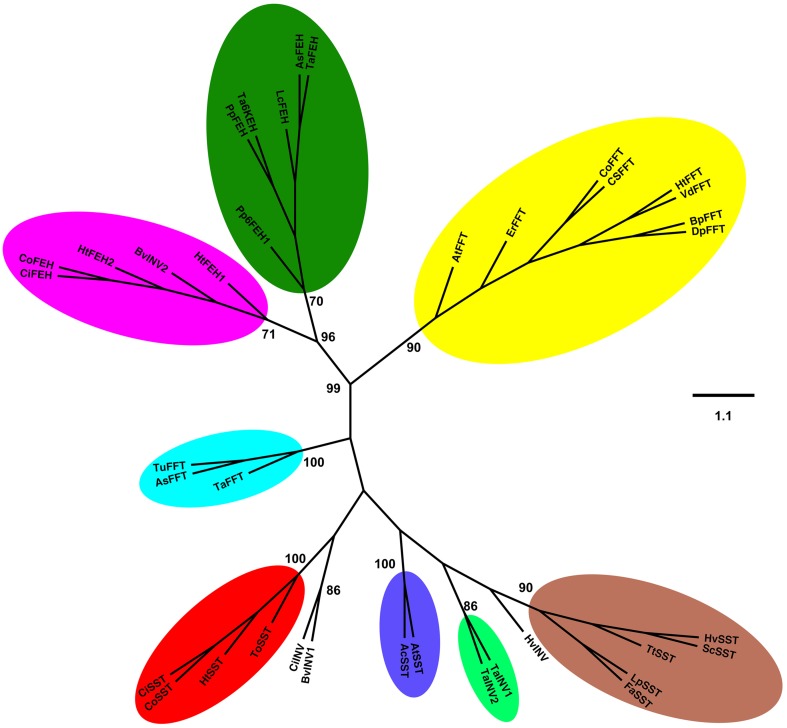
**Unrooted phylogenetic tree was inferred from the analysis of 38 amino acid sequences from plant 1-SST, 1-FFT, 1-FEH, and invertases, using the Neighbor-Joining method**. All positions containing alignment gaps and missing data were eliminated only in pairwise sequence comparisons. GenBank accession numbers are presented in parentheses. **Sucrose:sucrose 1-fructosyltransferase (1-SST)**: CoSST, *Chrisolaena obovata* 1-SST (KM597067); CiSST, *Cichorium intybus* 1-SST (JQ346799); ToSST, *Taraxacum officinale* 1-SST (AJ250634); HtSST, *Helianthus tuberosus* 1-SST (AJ009757); AtSST, *Agave tequilana* 1-SST (DQ535031); AcSST, *Allium cepa* 1-SST (AJ006066); HvSST, *Hordeum vulgare* 1-SST (AJ567377); LpSST, *Lolium perenne* 1-SST (AY245431); ScSST, *Secale cereale* 1-SST (JQ728010); TtSST, *Triticum turgidum* 1-SST (EU981912); FaSST, *Festuca arundinacea* 1-SST (AJ297369). **Fuctan:fructan 1-fructosyltransferase (1-FFT)**: CoFFT, *C. obovata* 1-FFT (KM597068); HtFFT, *H. tuberosus* 1-FFT (AJ009756); VdFFT, *Viguiera discolor* 1-FFT (AJ811625); CsFFT, *Cynara scolymus* 1-FFT (AJ000481); ErFFT, *Echinops ritro* 1-FFT (AJ811624); BpFFT, *Bellis perennis* 1-FFT (AJ811697); DpFFT, *Doronicum pardalianches* 1-FFT (AJ811696); AtFFT, *A. tequilana* 1-FFT (EU026119); TaFFT, *Triticum aestivum* 1-FFT (AB088410); AsFFT, *Aegilops searsii* 1-FFT (EU981914); TuFFT, *Triticum urartu* 1-FFT (EU981913). **1-Fructan exohydrolase (1-FEH)**: CoFEH, *C. obovata* 1-FEH(AM231149); CiFEH, *C. intybus* 1-FEHIIa (AY323935.1); HtFEH1, *H. tuberosus* 1-FEH1 (KJ946352); HtFEH2, *H. tuberosus* 1-FEH2 (KJ946353); PpFEH1, *Phleum pratense* 6-FEH1 (AB583555); PpFEH2, *Poa pratensis* 1-FEH (GU228510); LcFEH, *Leymus chinensis* 1-FEH (FJ178114); AsFEH, *Aegilops speltoides* 1-FEH (FJ184993); TaFEH1, *T. aestivum* 1-FEH (AJ508387); TaFEH2, *T. aestivum* 6-KEH (AB089271). **Vacuolar invertases (INV)**: CiINV, *C. intybus* vINV (AJ419971); TaINV1, *T. aestivum* vINV1 (AJ635225); TaINV2, *T. aestivum* vINV2 (AF069309); HvINV, *Hordeum vulgare* vINV (JQ4111256); BvINV1, *Beta vulgaris* vINV (XP_010676174). **Cell wall invertase (INV)**: BvINV2, *Beta vulgaris* cwINV (AJ277458) Bootstrap values for 1000 replicates are indicated as percentages (higher than 70%) along the branches.

An unrooted phylogenetic tree was built with the deduced amino acid sequences of *1-SST, 1-FFT* and *1-FEH* from *C. obovata* and other fructan accumulating species, and some invertases (Figure 1). The phylogenetic tree showed a clear separation between 1-SST, 1-FFT, 1-FEH, and invertases. Two distinct subgroups can be distinguished for 1-FEH, one containing sequences from Asteraceae and the other from Poaceae. Within the 1-SSTs, three subgroups can be differentiated, one consisting of Poaceae sequences, the other with sequences from Asteraceae, which includes *C. obovata* 1-SST, and a third small group with only two 1-SST sequences, from *A. tequilana* and *Allium cepa*. Vacuolar invertases from wheat and barley grouped with 1-SST from Poaceae while the vacuolar invertases from sugar beet and chicory grouped near the Asteraceae 1-SST. 1-FFT sequences formed a major subgroup including all Asteraceae 1-FFTs and one 1-FFT from *A. tequilana*, whereas the Poaceae 1-FFTs grouped together. As expected, the phylogenetic tree reveals that *C. obovata* 1-SST and 1-FFT are homologous to eudicotyledons rather than monocotyledons.

### Effects of different carbon sources on fructan metabolism

**Figure 2:**
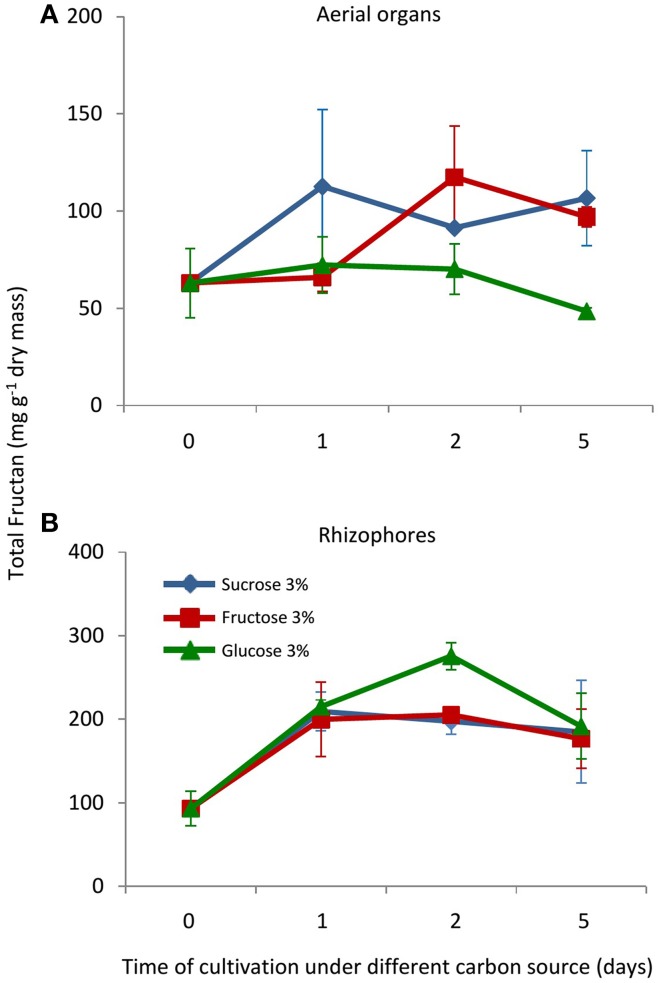
**Total fructan in plants of ***Crysolaena obovata*** cultured ***in vitro*** without a carbon source for 7 days (day 0) and subsequently transferred to different carbon sources (sucrose, fructose, or glucose 3%) for 5 days. (A)** Aerial organs, **(B)** Rhizophores. Values are means ± SE (*n* = 3). Means do not differ statistically by ANOVA.

**Figure 3:**
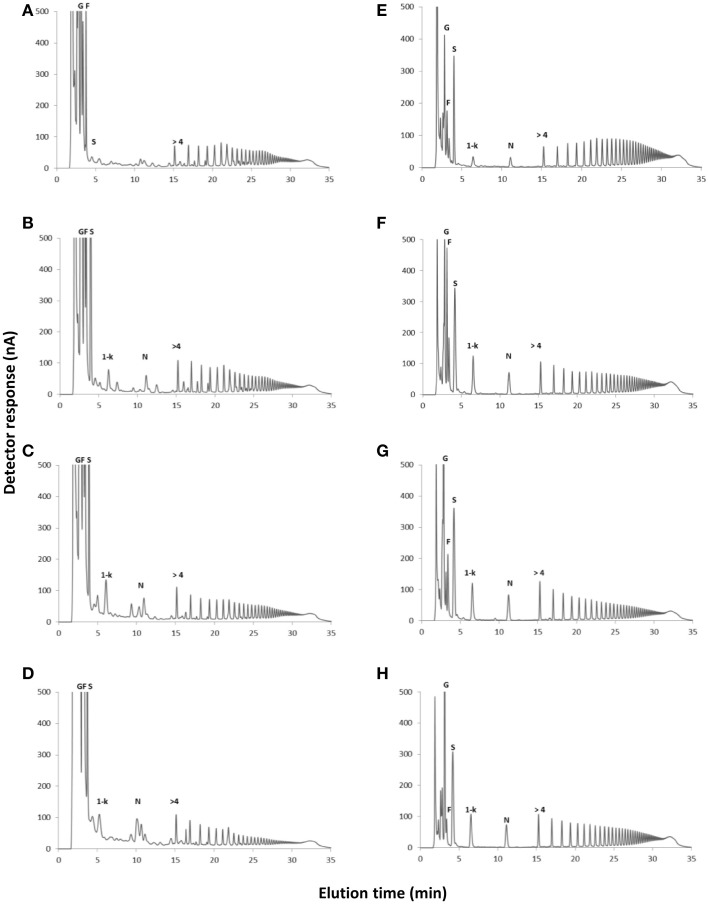
**HPAEC/PAD profiles of soluble carbohydrates extracted from plants of ***Crysolaena obovata*** cultured ***in vitro*** without a carbon source for 7 days (Control) and subsequently transferred to different carbon sources for 5 days**. Aerial organs: **(A)** Control, **(B)** Sucrose, **(C)** Fructose, **(D)** Glucose; Rhizophores: **(E)** Control, **(F)** Sucrose, **(G)** Fructose, **(H)** Glucose. G, glucose; F, fructose; S, sucrose; 1-k, 1-kestotriose; N, 1,1-kestotetraose; >4, fructans with DP higher than 4.

In control plants, cultivated in a medium free of carbon, total fructan content was 63.02 and 93.4 mg g^−1^ dry mass in aerial organs and rhizophores, respectively (Figure 2). Although values were not statistically different, fructan contents tended to increase after the transfer of plants to culture media supplemented with sucrose (Suc), fructose (Fru), or glucose (Glc), especially in rhizophores. HPAEC/PAD analysis of soluble sugars of aerial organs and rhizophores revealed the inulin homologous series, with similar profiles in all treatments, including an increase in medium DP fructans in the rhizophore of plants under different carbon source. When compared to the rhizophore, the aerial organs presented higher proportion of glucose, fructose and sucrose (Figure 3). Aerial organs also presented intermediate non-identified peaks neighboring 1-kestotriose and 1-kestotetraose, and others between higher DP components of the inulin series.

**Figure 4:**
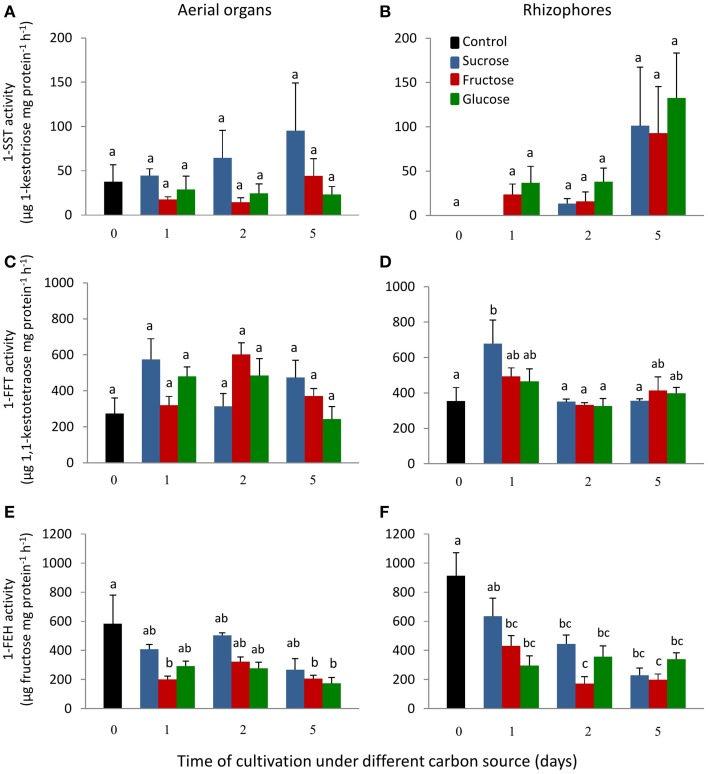
**1-SST, 1-FFT and 1-FEH activities in plants of ***Chrysolaena obovata*** cultured ***in vitro*** without a carbon source for 7 days (day 0) and subsequently transferred to different carbon sources (sucrose, fructose, or glucose 3%) for 5 days**. Aerial organs—**(A,C,E)**; Rhizophores—**(B,D,F)**. Values are means ± SE (*n* = 3). Letters compare same carbon source between days of cultivation within each plant organ (*P* < 0.05).

**Figure 5:**
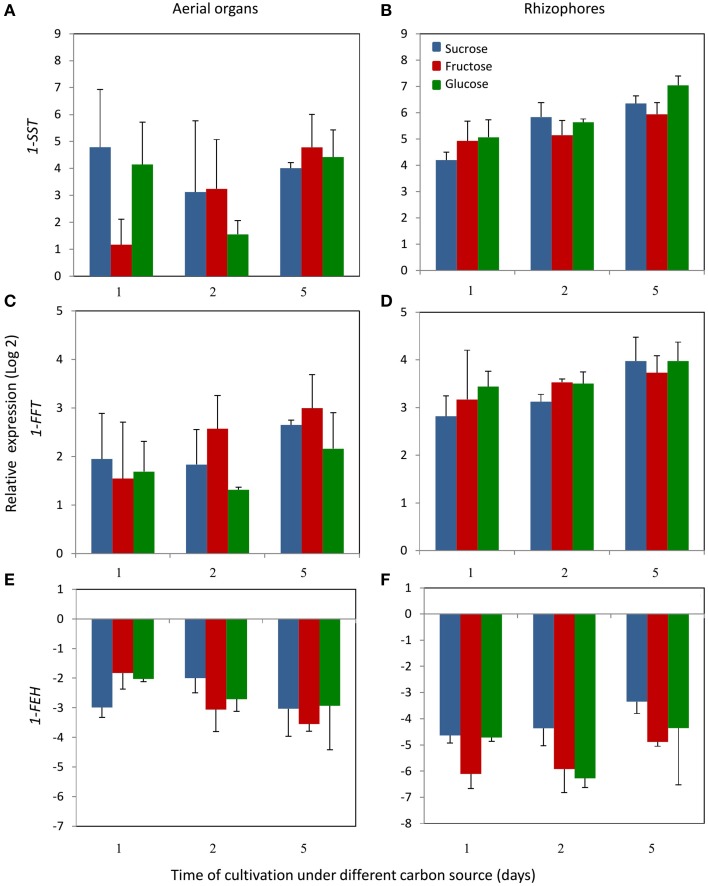
**Relative expression levels of ***1-SST, 1-FFT*** and ***1-FEH*** in plants of ***Chrysolaena obovata*** cultured ***in vitro*** without a carbon source for 7 days and subsequently transferred to different carbon sources (sucrose, fructose, or glucose 3%) for 5 days, using the elongation 1-alpha factor (***EF***) as reference gene**. Aerial organs—**(A,C,E)**; Rhizophores—**(B,D,F)**. Values are means (±SD) of two biological replicates with four technical replicates.

1-SST activity in the aerial organs of plants treated with fructose or glucose was similar to the control plants, while in plants under sucrose treatment, the activity showed a tendency of increase with time of cultivation (Figure 4A). In control plants, rhizophores did not present 1-SST activity; however, following the transfer to medium containing different carbon sources, the activity was detected from the first day of fructose and glucose treatments and from the second day under sucrose treatment. After 5 days, 1-SST activity in rhizophores reached the highest values, which were similar for all treatments, 101.2, 92.6, and 132.6 μg product mg protein^−1^ h^−1^ for sucrose, fructose and glucose medium, respectively (Figure 4B). *1-SST* relative expression in the aerial organs indicated increases of 4.0-fold (Suc), 4.8-fold (Fru), and 4.4-fold (Glc) when compared to control, after 5 days of cultivation (Figure 5A). The expression level of *1-SST* in rhizophores also increased with time of cultivation, presenting on the fifth day of culture, values 6.3-fold (Suc), 5.9-fold (Fru), and 7.0-fold (Glc) higher than control plants (Figure 5B).

1-FFT activity showed no changes in aerial organs or rhizophores over time, under the different carbon sources (Figures 4C,D). The relative expression of *1-FFT* increased with time after the transfer to medium with a carbon source. After 5 days of culture, the relative expression in aerial organs increased 2.7-fold (Suc), 3.0-fold (Fru), and 2.2-fold (Glc) while in rhizophores the increases were 4.0-fold (Suc), 3.7-fold (Fru), and 4.0-fold (Glc) that of the control (Figures 5C,D).

1-FEH showed the highest activity in control plants, 584.3, and 912.6 μg product mg protein^−1^ h^−1^ in aerial organs and rhizophores, respectively. However, the transference of plants to culture media containing the carbon sources resulted in the decrease of this activity, more markedly under fructose treatment (Figures 4E,F). The expression profile was in accordance with the activity assayed, with higher*1-FEH* transcript accumulation in the aerial organs and rhizophores of control plants subjected to carbon deficit. When transferred to media containing sucrose, fructose or glucose, the down-regulation of *1-FEH* was observed in aerial organs and rhizophores (Figures 5E,F). Fructose was more effective in the inhibition of *1-FEH* expression in rhizophores, showing a 6.1-fold (1st day), 5.9-fold (2nd day), and 4.9-fold (5th day) decrease of transcription when compared to control plants, excepting for the second day, when glucose showed a higher effect on inhibition of gene expression (6.2-fold).

Relative expression analysis of all genes presented similar patterns of expression when normalized by another reference gene (*18S*) (Figure S1).

### Effects of different sucrose concentrations on fructan accumulation

**Figure 6:**
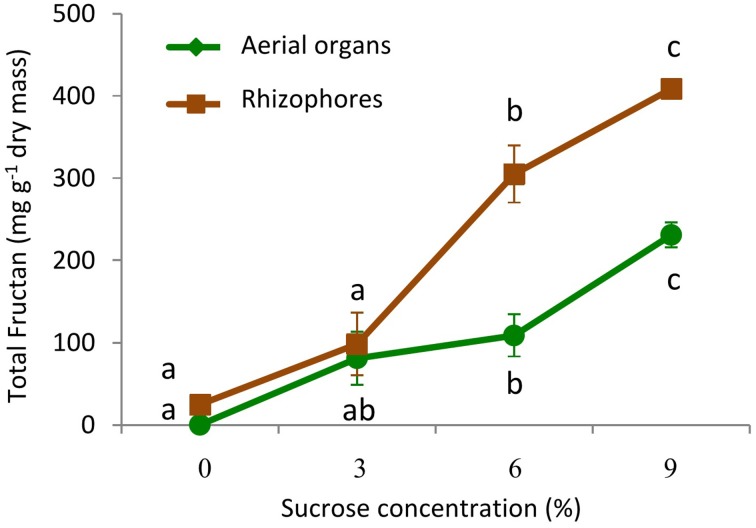
**Total fructan in plants of ***Crysolaena obovata*** cultured ***in vitro*** for 30 days under different sucrose concentrations (0, 3, 6, or 9%)**. Values are means ± SE (*n* = 4). Letters compare means at different sucrose concentrations within each plant organ (*P* < 0.05).

Growing plants for 30 days in culture medium without sucrose (0% Suc) led to the intensive consumption of plant reserves and to marked decrease in total fructans, from 72.5 to 0.16 mg g^−1^ dry mass in aerial organs, and from 108.6 to 24.8 mg g^−1^ dry mass in rhizophores (Figure 6). When transferred to culture media containing increasing sucrose concentrations (3, 6, and 9% Suc), fructan contents increased significantly in both aerial and underground organs, but more markedly, in the last ones, attaining in these organs, 408.5 mg g^−1^ dry mass under 9% Suc (Figure 6). Rhizophores and aerial organs from plants cultivated in all sucrose treatments presented the regular homologous inulin series (data not shown).

**Figure 7:**
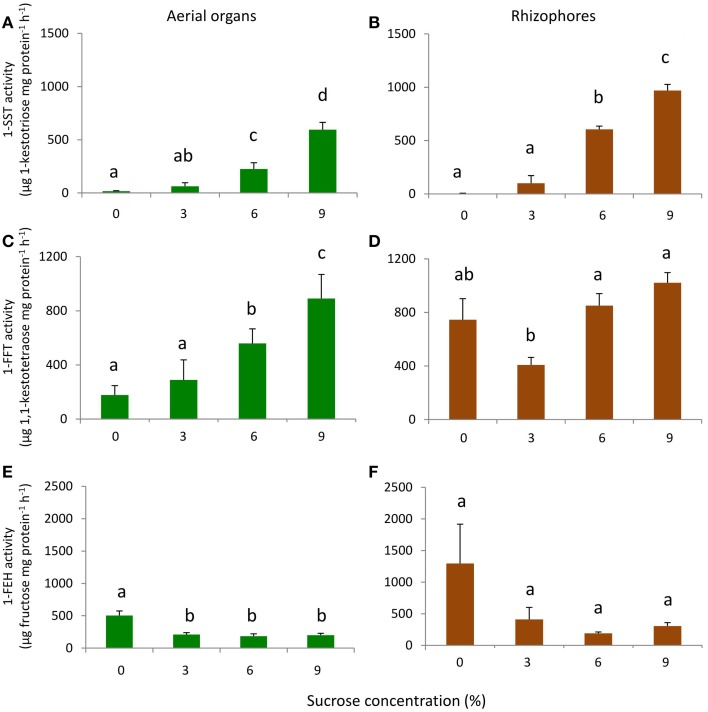
**1-SST, 1-FFT, and 1-FEH activities in plants of ***Chrysolaena obovata*** cultured ***in vitro*** for 30 days at different sucrose concentrations (0, 3, 6, or 9%)**. Aerial organs—**(A,C,E)**; Rhizophores—**(B,D,F)**. Values are means ± SE (*n* = 3). Letters compare means at different sucrose concentrations (*P* < 0.05).

1-SST showed a similar pattern of activity in aerial organs and rhizophores, with activity close to zero in the absence of sucrose, and increasing linearly with the increase of sucrose concentration. In 9% Suc, 1-SST activity was 595.4 (aerial organs) and 969.6 (rhizophores) μg product mg protein^−1^ h^−1^, values respectively 34 and 334 times higher than those detected in 0% Suc (Figures 7A,B).

The aerial organs showed a gradual increase in 1-FFT activity with the increase in sucrose concentration. In 9% Suc, the activity was 5 times higher than in 0% Suc. A different pattern of activity was observed in rhizophores, in which the activity in 0% Suc was higher than in 3% Suc, however, starting from this concentration, a pattern of activity similar to that of aerial organs was observed (Figures 7C,D).

In 0% Suc, values of 1-FEH activity were 504.3 and 1294.2 μg product mg protein^−1^ h^−1^ in aerial organs and rhizophores, respectively. With sucrose supplementation, 1-FEH activity decreased on average 2.5-fold in aerial organs and 4.2-fold in rhizophores, independently of the sucrose concentration used (Figures 7E,F).

## Discussion

Despite the importance of *Chrysolaena obovata* as a model species for understanding fructan metabolism *in vitro* and *in vivo*, only cDNAs coding for FEHs from this Cerrado species have been cloned (Asega et al., 2008). To further evaluate the relative expression of all fructan metabolism genes in response to different carbon sources, and since no genomic information is available for *C. obovata*, partial *1-SST* and *1-FFT* cDNA sequences were isolated. Sequence and phylogenetic analyses confirmed that *Co1-SST* and *Co1-FFT* are the *C. obovata* orthologs of the well functionally characterized genes involved in fructan synthesis in other Asteraceae species. Our results are in accordance with other phylogenetic analyses of *1-SST* and *1-FFT*, which grouped preferably with sequences from Asteraceae instead of Poaceae (Van den Ende et al., 2002), and are consistent with the hypothesis of polyphyletic origin of the genes involved in fructan synthesis in plants (Hendry and Wallace, 1993).

The pre-existing complete cDNA sequence of *Vh1-FEH* (Asega et al., 2008) and the partial sequences herein acquired, clearly enabled the expression analyses of fructan metabolism genes in plants of *C. obovata*, although the function of new sequences have not been tested in a heterologous system. To our knowledge, there is little information available on genes responsible for inulin synthesis in native Cerrado species (Van den Ende et al., 2005), and more efforts should be done in the future to isolate the complete sequence of these genes and to experimentally confirm its function.

The low fructan content observed in rhizophores of control plants indicates fructan mobilization to provide energy and carbon skeletons under sugar starving, in accordance with the highest 1-FEH activity and expression detected at this point. Fructan mobilization under low carbon input has been already demonstrated for *C. obovata* under greenhouse and field conditions (Asega and Carvalho, 2004; Asega et al., 2011; Rigui et al., 2015).

Exogenous sugar supply with sucrose, fructose or glucose induced the activity and expression of 1-SST and 1-FFT in rhizophores and repressed the activity and expression of 1-FEH. The increase in fructosyltransferases clearly leads to an accumulation of 1-kestotriose and medium DP fructan, showing a quick shift from source to sink organ independent of the carbon source. This confirms that the role of rhizophores as sink organs, described for *C. obovata* cultivated under optimal conditions of light, nutrients and water (Carvalho and Dietrich, 1993; Rigui et al., 2015), is maintained in *in vitro* condition.

The concomitant increase in the activity and relative expression of 1-SST and 1-FEH from *C. obovata* indicate a transcriptional regulation of fructan metabolism genes, as previously shown for *Festuca arundinacea* (Lüscher et al., 2000), *Hordeum vulgare* (Wang et al., 2000), and *Taraxacum officinale* (Van den Ende et al., 2000).

On the other hand, since changes in 1-FFT activity and transcriptional profile were slightly distinct, we cannot exclude a post-transcriptional regulation mechanism of 1-FFT or also the existence of different isoforms with distinct expression profiles. In this work, only one isoform of 1-FFT was isolated from *C. obovata* using degenerated primers, and specific primers assayed the expression level of this isoform, as confirmed by single melting curves. Reports of more than one 1-FFT isoform are still scarce for inulin-accumulating species, with only *T. officinale* having two isoforms of 1-FFT isolated (AJ829549, *FFT1*, and AJ829550, *FFT2*). Surprisingly, no *1-FFT* gene was found in the transcriptome of *A. tequilana*, suggesting this gene can be less expressed than 1-SST in the tissues and conditions used (Simpson et al., 2011).

Opposing activities and expression profiles of 1-SST and 1-FEH were detected, showing that in *in vitro* condition the temporal control of fructan-metabolizing enzymes in the vacuole is similar to that observed for plants of *C. obovata* growing in natural condition (Asega and Carvalho, 2004; Portes and Carvalho, 2006; Oliveira et al., 2010; Rigui et al., 2015). This is consistent with the hypothesis of a single regulatory mechanism, but with opposite effect, for these two genes (Wagner and Wiemken, 1986; Marx et al., 1997).

Comparative effects of sucrose, glucose and fructose in fructan metabolism showed that all carbon sources tested could considerably affect enzyme activities and gene expression under a few days of carbon supply, since a similar response pattern was observed, with the exception of 1-SST in aerial organs. While 1-SST activity was induced in aerial organs already 1 h after carbon supply, a lag phase was observed for 1-SST activity in rhizophores. This difference may be due to the preferential translocation of sucrose into the phloem, compared to fructose and glucose, which is promptly metabolized in the strongest sinks, the aerial organs, serving as substrate for fructan synthesis. As already shown by Trevisan et al. (2014), rhizophores of *in vitro* plants present a very limited growth and function as poor sinks, when compared to greenhouse plants, delaying the start of fructan synthesis from sucrose.

Most organisms have developed a sensing mechanism and signaling cascade to respond to the availability of different sugars. Exogenously supplied hexoses can be rapidly transformed in sucrose, and sucrose can be broken into hexoses, making it difficult to discriminate between sucrose, fructose and glucose signaling (Maleux and Van den Ende, 2007). Glucose and sucrose-responsive elements were found in the promoter region of the *FEHIIa* gene from *C. intybus*, suggesting the importance of these sugars in the regulation of fructan metabolism (Michiels et al., 2004).

Concerning the effect of different sugars, fructose acts as a more effective inhibitor of fructan hydrolysis in *C. obovata*. However, fructose inhibition of 1-FEH activity and expression is probably related to feedback sugar repression, since fructose is the main product of inulin degradation. Lothier et al. (2010) suggest that regulation of fructan mobilization in *L. perenne* is dependent of glucose sensing, since fructose supply led to a weaker inhibition of FEH activity when compared to glucose. Experiments with *C. intybus* hairy roots cultures also showed distinct effects of glucose and fructose on *1-SST* and *1-FFT* expression and inulin accumulation. A strong induction of fructosyltransferases transcript accumulation was observed only with sucrose or fructose as carbon source, whereas glucose was less efficient (Kusch et al., 2009). For the levan accumulating species barley and wheat, the highest induction of fructan synthesizing enzymes was obtained with sucrose, while glucose and fructose were also able to induce fructan synthesis, but in a lesser extent than sucrose (Müller et al., 2000; Noël et al., 2001).

The supplementation with increasing sucrose concentration induced inulin accumulation at values similar to that observed for plants grown in a greenhouse (607.2 mg g^−1^ dry mass), with a linear increase up to 9% of sucrose (262 mM), suggesting that sucrose can be an adequate carbon source for the *in vitro* production of this compound. In the presence of sucrose, 1-FEH activity was inhibited regardless of the concentration used, whereas 1-SST activity increased gradually with the increase in sucrose concentration. A significant inhibition of 1-FEH activity by sucrose was previously reported for *C. obovata* in much lower sucrose concentrations, ranging from 1 to 10 mM (Asega et al., 2008), as well as for *H. tuberosus* (Marx et al., 1997), *Triticum aestivum* (Van den Ende et al., 2003) and *L. perenne* (Lothier et al., 2007, 2014), which are inhibited by sucrose at concentrations of up to 40 mM. The similar inhibition profile of 1-FEH activity observed at the three sucrose concentrations employed in this work (87, 175, and 262 mM), suggest that the enzyme attained the highest percentage of inhibition in lower concentrations than the ones supplied herein. On the other hand, since low photosynthetic rates are commonly measured in plants grown *in vitro* (Grout, 1988), the absence of sucrose led to the consumption of fructan reserves, with the highest activity of hydrolysis and inhibition of fructan synthesis being measured after 30 days of starvation.

Finally, the distinct pattern observed for 1-SST and 1-FFT enzymes in rhizophores suggest a mechanism of differential regulation of these genes in *C. obovata*, in contrast with the results obtained when *A. tequilana* and *A. inaequidens* plants were transferred to culture medium supplemented with increasing sucrose concentrations from 3 to 8% (Súarez-González et al., 2014). For these species, a similar pattern of response to exogenous sucrose, with increased expression of *1-SST* and *1-FFT* genes, was described. Inhibitors of Ca^2+^ signaling and protein kinases/phosphatases also modulated the expression of *1-SST* and *1-FFT* in a similar manner, suggesting a common regulatory mechanism for both enzymes in *C. intybus* (Kusch et al., 2009).

Still for *C. intybus*, the response to a specific sugar source seems to be linked to nitrogen supply. The transfer from a medium containing 3% sucrose to a high carbon/low nitrogen medium induced *1-SST* and *1-FFT* expression and fructan accumulation and the opposite response was observed when plants were transferred back to a standard medium (Kusch et al., 2009). For *C. obovata*, the induction of fructan synthesis under high carbon is independent of nitrogen status, since high nitrate (16.9 mM) and ammonium (13.1 mM) concentrations were used in the culture medium. Although both species belong to the Asteraceae family and accumulate inulin in its underground reserve organs, the results indicate a distinct modulation of fructan metabolism by sugars. However, additional experiments have to be performed to determine whether these responses could be “species-specific” or related to differences between the two experimental systems.

In any case, this study demonstrated that *C. obovata in vitro* culture can be successfully used for investigation of fructan metabolism regulation by exogenous factors, since an excellent correlation was observed between *in vivo* and *in vitro* plants. The positive effects of different carbon sources on fructan accumulation opens up the possibility of further adapting *C. obovata in vitro* cultures for large-scale inulin production.

## Author contributions

FT, MC, and MG designed the research. FT conducted all the experiments and performed the statistical analysis. VO gave support for carbohydrate and enzymatic analyses. FT, VO, MC, and MG analyzed the data and wrote the paper. All authors have read, revised and approved the final manuscript.

### Conflict of interest statement

The authors declare that the research was conducted in the absence of any commercial or financial relationships that could be construed as a potential conflict of interest.

## Abbreviations

1-FEH: fructan exohydrolase
1-FFT: fructan:fructan fructosyltransferase
1-SST: sucrose:sucrose fructosyltransferase
18S: 18S ribosomal RNA
EF: Elongation 1-alpha factor
HPAEC/PAD: high performance anion exchange chromatography coupled to pulsed amperometric detector
MS: Murashige & Skoog culture medium
qRT-PCR: quantitative reverse transcription polymerase chain reaction.
