# Supplementary material for: Effects of different carbohydrate sources on fructan metabolism in plants of Chrysolaena obovata grown in vitro
Source: Front Plant Sci. 2015 Sep 7;6:681. doi: 10.3389/fpls.2015.00681 (PMC4561353; doi:10.3389/fpls.2015.00681)
Supplement: Supplementary file 1 [file Table1.PDF]

Table 1. Degenerated primers designed for isolation of *C. obovata 1-SST*, *1-FFT* and *EF* partial cDNAs.

**Gene**	**Primer sequence**	**Fragment size (bp)**	**Tm (◦C)**
1-SST	F 5’- GACATGATCAACTGGTTCCATCTA- 3’	384	60
	R 5’- ACACATTCCCACATACCAGTATG - 3’		59
1-FFT	F 5’- TGTTGGAYGAGCCGTTGCAYTC - 3’	561	68
	R 5’- TTGCAACKATGTCCAACTGYGTAG -3’		64
EF	F 5’- AACCACCGGTCCACTTGATCTAC - 3’	464	60
	R 5’- ATGATGAAACTTCCTTCACGATTT - 3’		60
