# Supplementary figures and images for: Effects of different carbohydrate sources on fructan metabolism in plants of Chrysolaena obovata grown in vitro
Source: Front Plant Sci. 2015 Sep 7;6:681. doi: 10.3389/fpls.2015.00681 (PMC4561353; doi:10.3389/fpls.2015.00681)

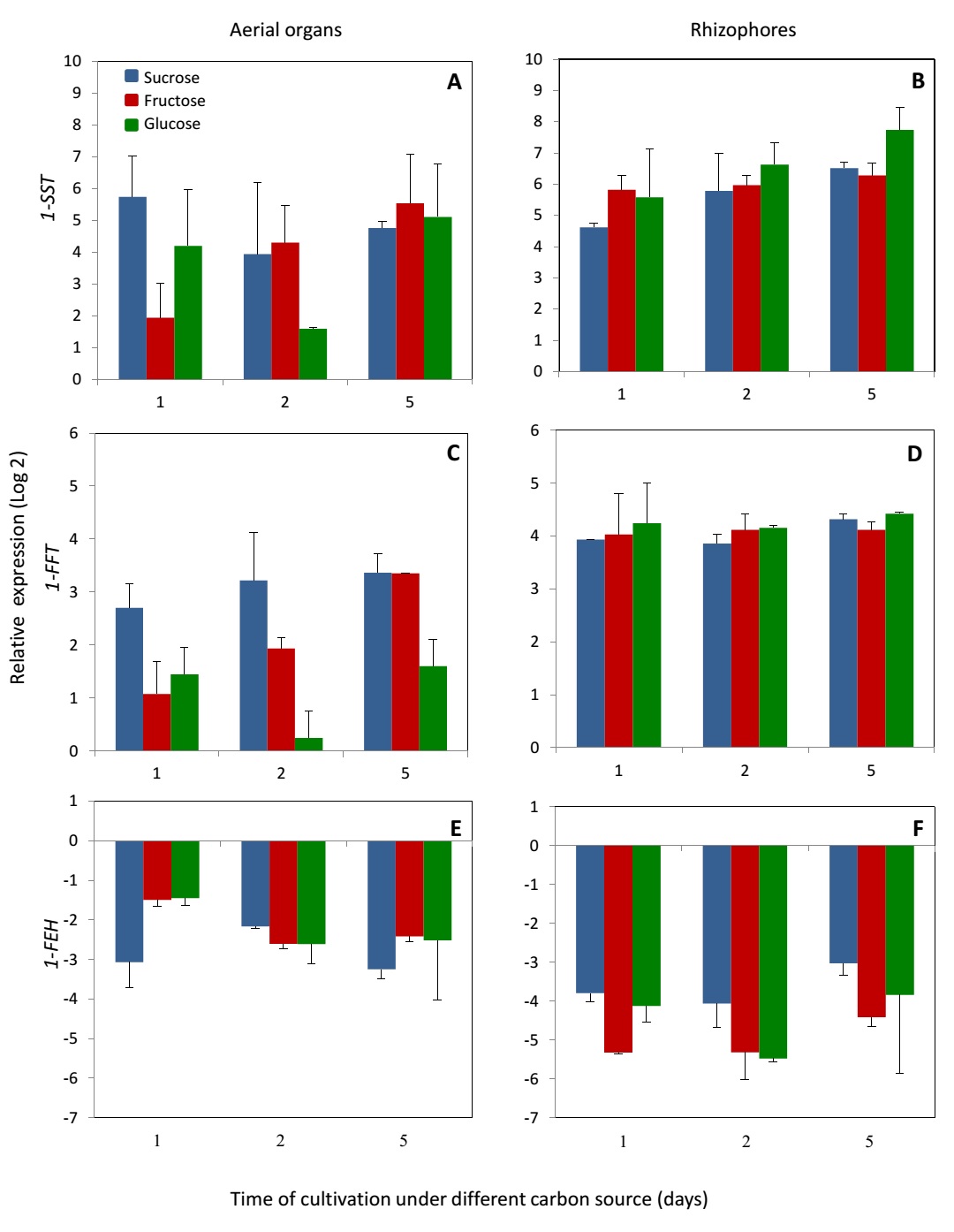

Supplement: Supplementary file 2 [file Image1.JPEG]
